# A New Colorimetric Test for Accurate Determination of Plastic Biodegradation

**DOI:** 10.3390/polym15102311

**Published:** 2023-05-15

**Authors:** Valérie Mattelin, Lennert Verfaille, Kankana Kundu, Stefaan De Wildeman, Nico Boon

**Affiliations:** 1 Center for Microbial Ecology and Technology (CMET), Ghent University, 9000 Ghent, Belgium; 2 B4Plastics, IQ Parklaan 2A, 3650 Dilsen-Stokkem, Belgium; 3 Center for Advanced Process Technology for Urban Resource Recovery (CAPTURE), 9000 Ghent, Belgium

**Keywords:** plastic, biodegradation test, colorimetry

## Abstract

As plastic waste is accumulating in both controlled waste management settings and natural settings, much research is devoted to search for solutions, also in the field of biodegradation. However, determining the biodegradability of plastics in natural environments remains a big challenge due to the often very low biodegradation rates. Many standardised test methods for biodegradation in natural environments exist. These are often based on mineralisation rates in controlled conditions and are thus indirect measurements of biodegradation. It is of interest for both researchers and companies to have tests that are more rapid, easier, and more reliable to screen different ecosystems and/or niches for their plastic biodegradation potential. In this study, the goal is to validate a colorimetric test, based on carbon nanodots, to screen biodegradation of different types of plastics in natural environments. After introducing carbon nanodots into the matrix of the target plastic, a fluorescent signal is released upon plastic biodegradation. The in-house-made carbon nanodots were first confirmed regarding their biocompatibility and chemical and photostability. Subsequently, the effectivity of the developed method was evaluated positively by an enzymatic degradation test with polycaprolactone with *Candida antarctica* lipase B. Finally, validation experiments were performed with enriched microorganisms and real environmental samples (freshwater and seawater), of which the results were compared with parallel, frequently used biodegradation measures such as O_2_ and CO_2_, dissolved organic carbon, growth and pH, to assess the reliability of the test. Our results indicate that this colorimetric test is a good alternative to other methods, but a combination of different methods gives the most information. In conclusion, this colorimetric test is a good fit to screen, in high throughput, the depolymerisation of plastics in natural environments and under different conditions in the lab.

## 1. Introduction

Biodegradation rates of conventional plastics are extremely low, even in optimized laboratory conditions [1,2,3,4]. This is due to the nature of plastics, but also to the (environmental) conditions. Plastics have, in general, a very low bioavailability, due to their solid and crystalline nature. Biological degradation of plastics is thus a selective process and dependent on the susceptibility of the polymer carbon backbone to microbial attack, as the polymer itself is too large to enter the cell [5,6,7]. Although plastic is a substrate with a high-energy content, even those types possessing well hydrolysable structural elements, such as amide and ester bonds, remain a poor growth substrate [1,6]. For polyesters, for example, the hydrolysis of the ester bonds is considered the rate-limiting step in both natural and engineered systems [8]. Due to the low bioavailability, the driver of biochemical evolution is also restricted [1]. Not only conventional plastics, that are resistant to microbiological action, but also plastics classified as biodegradable can have low biodegradation rates in some natural environments [9,10]. Especially in the marine environment, the lifetime of plastics can range from decades even to centuries [1]. For example, a polyhydroxyalkanoate (PHA) water bottle could take between 1.5 and 3.5 years to completely biodegrade in the marine environment (the biodegradation rate in marine environment for PHA is 0.04–0.09 mg/day/cm^2^) [11].

It is, however, of utmost importance to consider the conditions in which biodegradation experiments are conducted. Reported biodegradation rates of (biodegradable) plastics are very variable, due to variation in the natural environments, the experimental conditions and the test method used [12,13]. Because of the lack of understanding of all interdependent factors controlling degradation in natural environments, it also remains very difficult to predict the lifetime of biodegradable plastics [14]. Nevertheless, screening tests, which are used to quickly and reliably test whether a bioplastic would be biodegradable in a certain habitat in the long term, are very valuable for plastic biodegradation research and applications.

Many assays have been developed to study plastic biodegradation. Some methods are based on physical or chemical changes in the polymer structure, some on the elimination of small molecules and some on the growth properties of microorganisms. Not all methods assess complete biodegradation, i.e., from depolymerisation by extracellular enzymes to the mineralisation to CO_2_, but only one step of the process [4], for example, weight loss. Additionally, it remains difficult to distinguish between abiotic fragmentation into micro- and nanoplastics and complete biodegradation to CO_2_ [6]. Some frequently reported assays are weight loss, clearing zones, pH-stat titration, evaluation of material modifications by Fourier transformed infrared spectrometry (FTIR) or scanning electron microscopy (SEM), measurement of intermediate compounds by chromatography or dissolved organic carbon, CO_2_ evolution and O_2_ consumption [15,16,17]. Nowadays, the universally approved biodegradation tests are mainly based on CO_2_ measurements [13,18]. These test methods, however, require a minimum of three months incubation, have a low throughput and cannot discriminate between the degradation of the polymer itself or the degradation of (leached) additives or mono/oligomers [6,8,19,20]. Additionally, these standards test methods are unable to realistically predict biodegradability in the marine environment due to shortcomings in existing test procedures, as well as the absence of relevant standards for the majority of unmanaged aquatic habitats [13]. More sensitive methods, such as ^13^C or ^14^C-labeled synthetic polymers, provide essential and valuable data, but are hazardous, time-consuming, expensive and require specific equipment [6,16,21,22,23].

This research article focuses on a less frequently used but very valuable technique, namely, colorimetric assays. Colorimetric assays are reported in different applications, from on-site sensing strategies to detection of degradation, and have been confirmed in their easy feasibility and speed [19,24,25]. In this manuscript, the fluorescent substance is embedded in the polymer matrix, and the release of fluorescent signal in the medium gives information on depolymerisation, the rate-limiting step of biodegradation [26]. Colorimetric methods, based on the incorporation of fluorescent dyes or particles, lead to sensitive, in situ and real-time monitoring, which can be used in high throughput without expensive equipment. However, the used dyes may abiotically leach before biodegradation or give a fluorescent signal when embedded in the matrix [16,19]. Previously, fluorescent dyes such as nile blue [19] and methylene blue [24] have been used in colorimetric assays. However, concentrations from 0.100–10 mg/mL of these dyes result in microbial growth inhibition [27]. Additionally, biocompatible fluorescent dyes are often not very stable. For example, methylene blue is reduced by cell enzymes and returns colorless [28], fluorescein isothiocyanaat (FITC) decomposes in water [29] and nile blue undergoes spontaneous hydrolysis to nile red [30]. Therefore, the use of carbon nanodots as fluorescent material for colorimetric plastic biodegradation tests in natural environments is proposed here. 

Carbon nanodots have widespread applications in bioimaging, owing to properties such as biocompatibility, chemical and photostability and water-dispersibility [31,32]. They have been used in the synthesis of fluorescent hydrogels for different applications [33] such as sensing and removing rifampicin drug [34], selectively detecting Fe^3+^ [35] and adsorption and photodegradation of dyes from waste water [36]. Contrary to these applications aiming to retain a stable hydrogel, a recent study used carbon nanodots in hydrogels to monitor in vivo hydrogel biodegradation [19]. In this research, we take this one step further and present a colorimetric assay with the use of in-house made photostable, non-toxic carbon nanodots, to test biodegradation of different types of plastics in different environmental conditions. The in-house made carbon nanodots were characterised, and carbon nanodots-containing plastic pieces were used as substrates for a proof of concept by enzymatic degradation. Two case studies with microbial communities were executed to investigate the applicability of the colorimetric assay. The first case study was set up with an enriched seawater culture and the second with both freshwater from the Coupure (canal in Ghent, Belgium) and fresh seawater (sampled in Ostend, Belgium).

## 2. Materials and Methods

### 2.1. Media Preparation and Plastic Supply

The media used in this manuscript were ONR7a as described by Atlas (2004), marine broth (BD Difco™ Dehydrated Culture Media: Marine Broth 2216, Becton Dickinson, Franklin Lakes, NJ, USA) and mineral salts medium without carbon (MMC), adapted from Stanier et al. [37], by elimination of all carbon. For the mineral salts medium, 10 mL of a 1 M stock solution of Na_2_HPO_4_ and KH_2_PO_4_ was mixed with 3 mL of a 100 g/L stock solution of (NH_4_)_2_S0_4_, 5 mL of a 19.7 g/L stock solution of MgSO_4_, 5 mL of a 1.150 g/L stock solution of CaCl_2_·2H_2_O and 5 mL of a trace elements solution (containing 0.640 g/L ETDA, 0.230 g/L ZnSO_4_·7H_2_O, 0.550 g/L FeSO_4_·7H_2_O, 0.340 g/L MnSO_4_·H_2_O, 0.075 g/L CuSO_4_·5H_2_O and 0.025 g/L (NH_4_)_6_Mo_7_O_24_·4 H_2_O) and diluted in Instant OceanTM (35 g/L) to a total volume of 1 L.

The plastics used in the experiments were Poly(3-hydroxybutyrate-co-3-hydroxyhexanoate) (PHBH, Green Planet™ X331N, Kaneka biopolymers, Pasadena, TX, USA), Poly(butylene succinate-co-butylene adipate) (PBSA, PTT MCC Biochem BioPBS FD92), polylactic acid (PLA, Ingeo 6302D) and polycaprolactone (PCL, Perstorp CAPA 6800). Each plastic was dissolved in chloroform (5 wt%) and mixed with the in-house carbon nanodots (supplied by B4Plastics, Dilsen-Stokkem, Belgium), and subsequently, the solvent was evaporated by rotary evaporation. The concentration of the carbon nanodots for the enzymatic, enriched seawater culture and Coupure tests was 1.072 mg/g plastic; only for the seawater test, a 5 times higher concentration of 5.36 mg/g plastic was used.

### 2.2. Characterisation of the Carbon Nanodots as Fluorescent Biodegradation Indicator

All fluorescence measurements were performed in a 96-well microplate without lid, black, high binding, sterile (Greiner Bio-One, Kermsmünster, Austria) with a Tecan Infinite^®^ M200 PRO multiwell plate reader (Tecan, Männedorf, Switzerland) in three technical triplicates. 

### 2.3. Photobleaching of Carbon Nanodots

The carbon nanodots were incubated at room temperature (21 °C) in three replicates, one series exposed to natural daylight and one series incubated in the dark as a negative control. Fluorescence was followed for seven weeks with a Tecan Infinite^®^ M200 PRO multiwell plate reader (Tecan, Männedorf, Switzerland) and pH was measured with a InLab Flex-Micro pH electrode (Mettler-Toledo, Columbus, OH, USA).

### 2.4. Influence of pH on Fluorescence of Carbon Nanodots

Phosphate buffers varying in pH from 5 to 8 were used to analyse the influence of the pH on the fluorescence of the carbon nanodots. Fluorescence was measured with a Tecan Infinite^®^ M200 PRO multiwell plate reader (Tecan, Männedorf, Switzerland) and pH was measured before and after adding the carbon nanodots to the buffers (Mettler-Toledo, Columbus, OH, USA).

### 2.5. Toxicity of Carbon Nanodots

An enriched seawater culture (10^6^ cells/mL enriched on PHBH) was exposed to 1.34 × 10^−6^ mg/mL carbon nanodots, with marine broth (BD Difco™) as growth medium in three biological replicates. The samples were incubated at 21 °C at 300 rpm. The cell density was followed by continuous optical density (OD_600_) measurement (21 °C), performed with a Tecan Infinite^®^ 200 PRO Nano Quant multiwell plate reader (Tecan, Männedorf, Switzerland).

### 2.6. Bioavailability of the Carbon Nanodots

An enriched seawater culture, diluted in ONR7a to 10^6^ cells/mL, was incubated with carbon nanodots (1.34 × 10^−4^ mg/mL) as the only carbon source, in three biological replicates. The OD_600_ was measured continuously for seven days with the Tecan Infinite^®^ 200 PRO Nano Quant multiwell plate reader (Tecan, Männedorf, Switzerland). After the experiment, the remaining carbon nanodots were determined by measuring the fluorescence with the Tecan Infinite^®^ M200 PRO multiwell plate reader (Tecan, Männedorf, Switzerland).

### 2.7. Enzymatic Degradation Experiment

Erlenmeyers containing 50 mL of 0.1 M and 0.05 M phosphate buffer, 10% Candida antarctica lipase B (CALB) and 5 mg/mL fractionated PCL (containing 1.072 mg carbon nanodots/g plastic) submerged in the solution were prepared in biological triplicates. Controls were added for abiotic leaching of carbon nanodots (without CALB) as well as a negative control without PCL. The Erlenmeyers were incubated at 28 °C and 120 rpm. Fluorescence and pH were monitored, respectively, with the Tecan Infinite^®^ M200 PRO multiwell plate reader (Tecan, Männedorf, Switzerland) and the pH electrode InLab Flex-Micro (Mettler-Toledo, Columbus, OH, USA) until a plateau phase was reached after six days. 

### 2.8. Validation of Fluorescent Nanoparticles in a Biodegradation Assay

#### 2.8.1. Case Study 1: Enriched Seawater Culture (Originally Enriched on PHBH) in MMC Medium

Serum bottles were set up with 1 g/L of PCL, PLA, PBSA or PHBH (containing 1.072 mg carbon nanodots/g plastic) in mineral salts medium without carbon (MMC), in biological replicates. An enriched seawater culture, originally enriched on PHBH, was diluted to 10^6^ cells/mL and used as inoculum. As a negative control, autoclaved MMC medium without inoculum was used, with 160 mM NaN_3_, supplied as an antimicrobial chemical reagent, as described by Otte et al., 2018 [38]. One serum bottle contained only the seawater culture, but without plastic. Serum bottles were incubated at 20 °C, 100 rpm. For four weeks, fluorescence was monitored with the Tecan Infinite^®^ M200 PRO multiwell plate reader (Tecan, Männedorf, Switzerland). Validation of this assay was completed by performing dissolved organic carbon (DOC, Sievers™ 900 Series Global Analyser Solutions, Breda, The Netherlands) analysis (not for the negative control with NaN_3_), flow cytometry (described below, BD FACSVerse™, BD Biosciences, Erembodegem, Belgium) and gas chromatography (GC, Compact GC4.0, Global Analyser Solutions, Breda, The Netherlands, Chromeleon 7 software). Sampling occurred twice a week, except for DOC, which was sampled only once a week. Controls were sampled once a week and subjected to all analyses.

#### 2.8.2. Case Study 2: Environmental Sample: Coupure and Seawater

Coupure water was sampled on 3 March 2022 in Ghent, Belgium (51.052890 N, 3.709599 E). Seawater was sampled on 21 December 2022 (51.235889 N, 2.915930 E). For both environmental samples, serum bottles with 1 g/L PCL (containing 1.072 mg carbon nanodots/g plastic and 5.36 mg carbon nanodots/g plastic, respectively) were prepared, in biological triplicates. The serum bottles were closed with rubber stoppers and 10 v% headspace was kept. Negative controls contained either Coupure or seawater without plastics and with 160 mM NaN_3_ as an antimicrobial reagent. Pyruvate was supplied in 0.01 *w*/*v*%. For seven (seawater) and eight (Coupure water) weeks, biodegradation was followed by several methods: fluorescence was measured by Tecan Infinite^®^ M200 PRO multiwell plate reader (Tecan, Männedorf, Switzerland), the pH (InLab Flex-Micro, Mettler-Toledo, Columbus, OH, USA)), dissolved organic carbon (DOC, Sievers™ 900 Series Global Analyser Solutions, Breda, The Netherlands) analysis (not for NaN_3_), flow cytometry (staining according to section ‘Case Study 1’) (BD FACSVerse™, BD Biosciences, Erembodegem, Belgium and Attune NxT, Thermo Fisher Scientific, Waltham, MA, USA), gas chromatography (GC, Compact GC4.0, Global Analyser Solutions, Breda, The Netherlands, Chromeleon 7 software). Sampling occurred once a week.

### 2.9. Analytical Techniques

For both case studies, the following analytical techniques were used: flow cytometry, gas chromatography, pH and dissolved organic carbon analysis.

For flow cytometry measurements, sample aliquots were taken and diluted in sterile PBS (Merck/Sigma-aldrich, Burlington, MA, USA). The samples were stained with SYBR^®^ Green I combined with 50 × 20 mM propidium iodide (SGPI, Invitrogen, 100× diluted in 0.22 μm-filtered dimethyl sulfoxide) for 20 min at 37 °C in the dark, as described previously [39]. The cell concentration was measured with the flow cytometer BD FACSVerse™ Cell Analyser (BD Biosciences, Erembodegem, Belgium), equipped with a blue (488 nm), red (640 nm) and violet (405 nm) laser, and the Attune NxT flow cytometer (Fisher Scientific^TM^, Waltham, MA, USA), equipped with a blue (488 nm) and red (637 nm) laser, with PMT values at 180 for FSC, 230 for SSC, 325 for SG fluorescence and 400 for PI fluorescence.

The gas phase composition of the headspace was analysed with a Compact GC4.0 (Global Analyser Solutions, Breda, The Netherlands), equipped with a Molsieve 5A pre-column and Porabond Q column (CH_4_, O_2_, H_2_ and N_2_). Concentrations of gases (O_2_ and N_2_) were determined by means of a thermal conductivity detector, from which the CO_2_ concentration was calculated by the ideal gas law.

The pH was measured by using a InLab Flex-Micro, pH electrode (Mettler-Toledo, Columbus, OH, USA). and controller (consort C3020, Turnhout, Belgium), calibrated with commercially available pH buffers (Carl Roth, Belgium).

For dissolved organic carbon, samples were diluted in ultrapure water and filtered over 0.22 µm in AOC-free vials, prepared according to Hammes and Egli (2005) [40]. Samples were analysed in a Sievers 900 Series Total Organic Carbon Analyzer (GE Analytical instruments, PMT Benelux, Kampenhout, Belgium). The oxidizer flowrate was adjusted to the organic load of the sample. Samples were measured in three technical replicates.

### 2.10. Data Analysis

Cell concentrations were extracted from flow cytometric standard (FCS) format files using Phenoflow (v1.1.2) [41]. Gating was performed to count only the living cells. Data analysis was performed using statistical packages including vegan (v2.5.6) [42].

## 3. Results

### 3.1. Characterisation of Fluorescent Nanoparticles as Indicator for Plastic Biodegradation

Several characteristics of the in-house carbon nanodots were investigated for their use in colorimetric assays. First of all, the compound should not interfere with the bacterial metabolism, i.e., it should not be bioavailable and should not be toxic. Secondly, for colorimetric assays, it is important that the compound is photostable. Thirdly, as the pH can be variable during biodegradation, it is important that influence of the acidity on fluorescence is verified.

#### 3.1.1. Interactions between Nanoparticles and Bacteria

**Figure 1:**
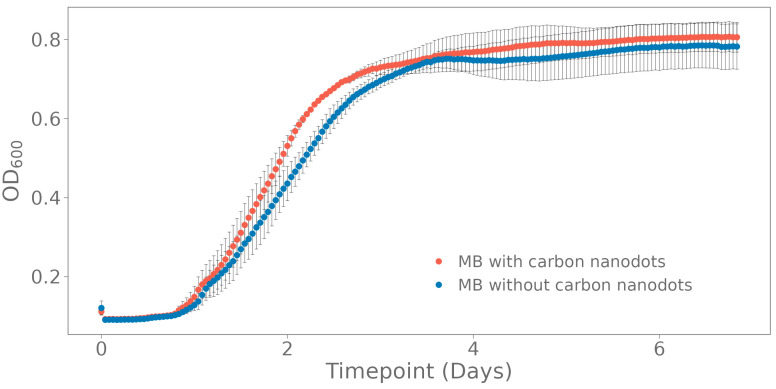
Carbon nanodots were added to an enriched seawater culture grown in marine broth (MB) to test their toxicity. Growth was measured by following OD_600_ over time. The standard deviation was calculated on three biological replicates.

The effect of the nanoparticles on the growth of an enriched seawater culture in marine broth was investigated by OD_600_ absorbance. The obtained growth curve shows a similar final cell concentration (0.8059 ± 0.762 for MB with carbon nanodots and 0.7823 ± 0.1102 for pure MB) and thus no negative effect on microbial growth (Figure 1).

**Figure 2:**
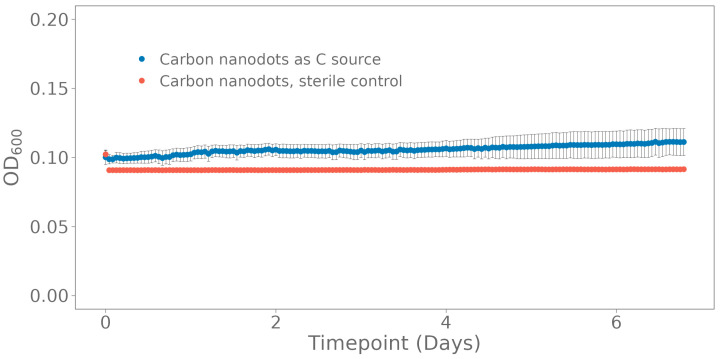
Bioavailability test with carbon nanodots for the enriched seawater culture in ONR7a medium, measured by following OD_600_ over time with carbon nanodots as the only carbon source. The standard deviation was calculated on three biological replicates.

Potential bioavailability of the nanoparticles was examined by incubation of the enriched seawater culture in ONR7a medium with the carbon nanodots as the only C source. The OD_600_ increased from 0.1029 ± 0.0643 to 0.1123 ± 0.0815 (Figure 2). The fluorescence of the carbon nanodots increased during the experiment from 6544 ± 728 to 4406 ± 629.

#### 3.1.2. Physicochemical Characteristics of the Nanoparticles

**Figure 3:**
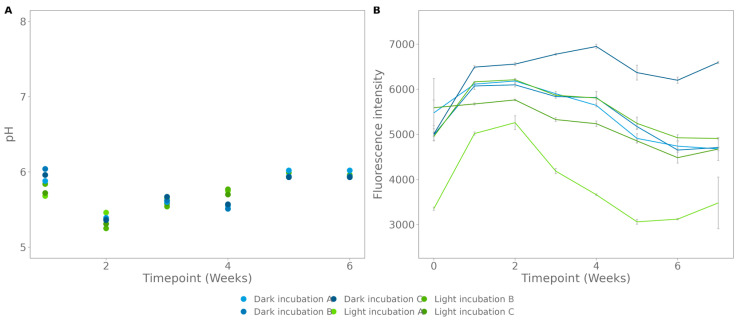
Photobleaching test for the carbon nanodots, measured by incubation in daylight and in the dark for 6 weeks: (**A**) pH, and (**B**) fluorescence; the standard deviation was calculated on three technical replicates.

The photostability of the compound was tested by incubation in both daylight and the dark. The pH fluctuated between 6.04 (replicate B, dark, week 1) and 5.25 (replicate B, light, week 3). The carbon nanodots were added to non-buffered, ultrapure water with an initial pH of 7.18 (Figure 3A), and they altered the pH during the course of the experiment. The fluorescence intensity was measured in triplicate, of which replicate B and C of the daylight incubation and A and B of the incubation in the dark follow the same fluctuating trend (Figure 3B). 

**Figure 4:**
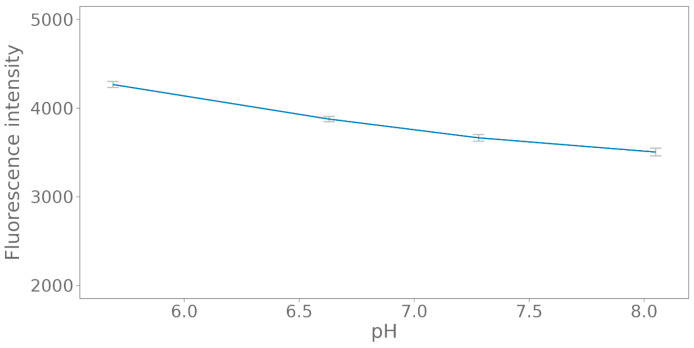
The influence of pH on the fluorescence of the carbon nanodots. The standard deviation was calculated on three replicates.

As the fluorescence pattern seems to be negatively correlated to the change in pH (Figure 3), the potential influence of pH on the fluorescent properties was tested in a relevant pH range for microbial degradation test setups (Figure 4). A negative correlation was observed from 4266 ± 59 at pH 5.69 to 3505 ± 75 at pH 8.05.

### 3.2. Proof of Concept: Enzymatic Degradation of PCL

**Figure 5:**
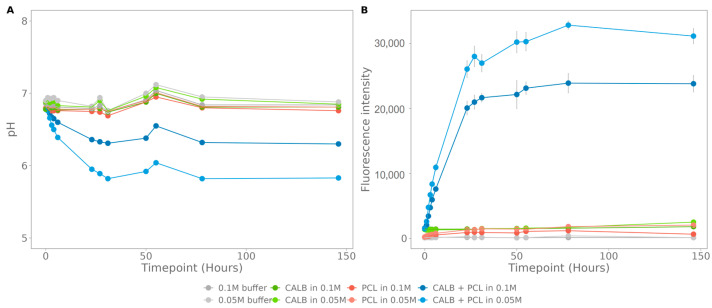
Enzymatic degradation of polycaprolactone (PCL) with *Candida antarctica* lipase B (CALB) in 0.1 M and 0.05 M phosphate buffer: (**A**) pH, and (**B**) fluorescence; the standard deviation was calculated on three technical replicates.

To validate the potential use in microbial degradation assays, polycaprolactone (PCL), with incorporated carbon nanodots was incubated with Candida antarctica lipase B (CALB), an enzyme proven to degrade PCL [43,44,45]. The pH for both phosphate buffers (0.1 M and 0.05 M) decreases to a plateau of, respectively, 6.3 and 5.83, confirming biodegradation (Figure 5A). The decrease in pH correlates with an increase in fluorescence for both buffered systems, with a plateau at 23 790 ± 734 and 31 115 ± 672, respectively (Figure 5B). The fluorescence corresponds to 22 ± 0.54% carbon nanodots that are released for the 0.1 M buffer and 28 ± 0.48% for the 0.05 M buffer (standard curve: Fluorescence = $2\times 10^{7}\times CNconcentration\left(\frac{mg}{mL}\right)+153.25$, R^2^ = 0.9999). The plateau, reached for both fluorescence and pH, was due to inactivity of the enzyme. The controls did not show an increased fluorescence or decreased pH.

### 3.3. Case Study 1: Degradation of Plastics by an Enriched Microbial Community

To assess the applicability of the colorimetric method for measuring biodegradation by microorganisms, incubations with an enriched seawater culture (initially enriched on PHBH) and PCL, PLA, PBSA and PHBH, containing carbon nanodots, were set up. Biodegradation was measured by fluorescence and compared with the following methods: flow cytometry, O_2_ consumption, CO_2_ respiration and dissolved organic carbon (DOC).

**Figure 6:**
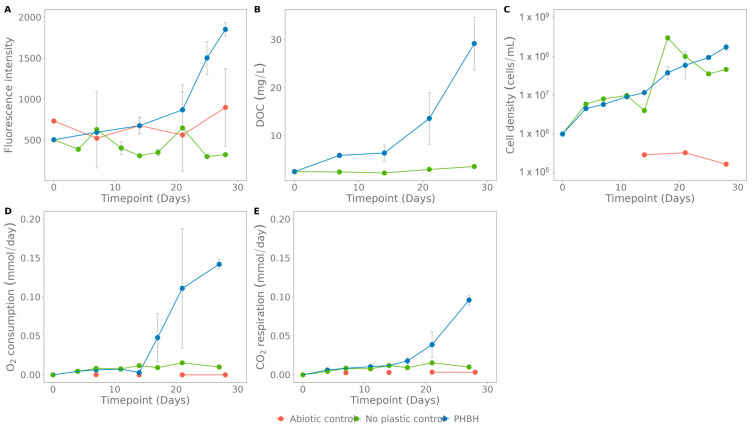
Assays for poly(3-hydroxybutyrate-co-3-hydroxyhexanoate) (PHBH) biodegradation with the enriched seawater culture: (**A**) fluorescence, (**B**) dissolved organic carbon, (**C**) cell density, (**D**) O_2_ consumption rate, and (**E**) CO_2_ respiration rate. The abiotic control contains only plastic and NaN_3_, the no-plastic control contains only the enriched culture in medium. The standard deviation was calculated on three biological replicates.

Biodegradation of PHBH, the plastic on which the culture was originally enriched, was confirmed by all performed analyses (Figure 6). First, up until day 14, mainly the cell density increases (Figure 6C). Afterwards, fluorescence, O_2_ consumption rate, CO_2_ respiration rate and DOC start increasing at a higher rate. The latter confirms the depolymerisation measured by fluorescence and the concomitant release of soluble oligo- and monomers and growth and respiration by the cells. The released fluorescence corresponds to 8.5 ± 0.2% released carbon nanodots and correspondingly 18.7 ± 0.3 mg plastic that is degraded.

**Figure 7:**
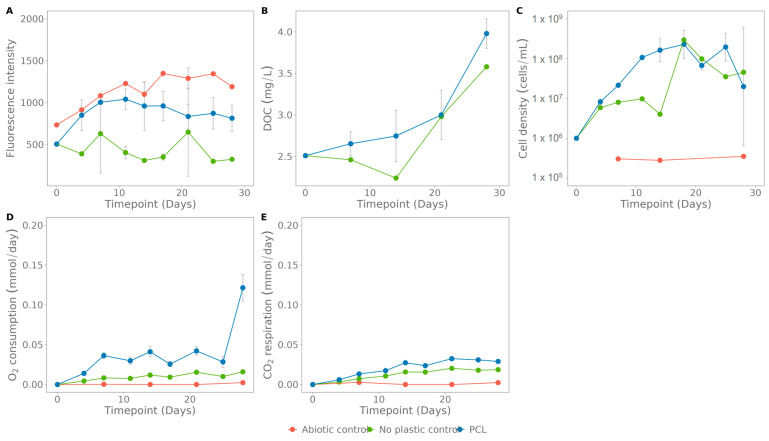
Assays for polycaprolactone (PCL) biodegradation with the enriched seawater culture: (**A**) fluorescence, (**B**) dissolved organic carbon, (**C**) cell density, (**D**) O_2_ consumption rate, and (**E**): CO_2_ respiration rate. The abiotic control contains only plastic and NaN_3_, the no-plastic control contains only the enriched culture in medium. The standard deviation was calculated on three biological replicates.

A similar set up was used to test biodegradation of PCL with the enriched community. In this case, the fluorescence at 28 days has similar levels as the negative control (824 ± 162 versus 854 for the negative control), indicating no biodegradation (Figure 7A). The other analyses show an increase over time in DOC, cell density and CO_2_ respiration rate, comparable to the control without plastic (Figure 6).

**Figure A1:**
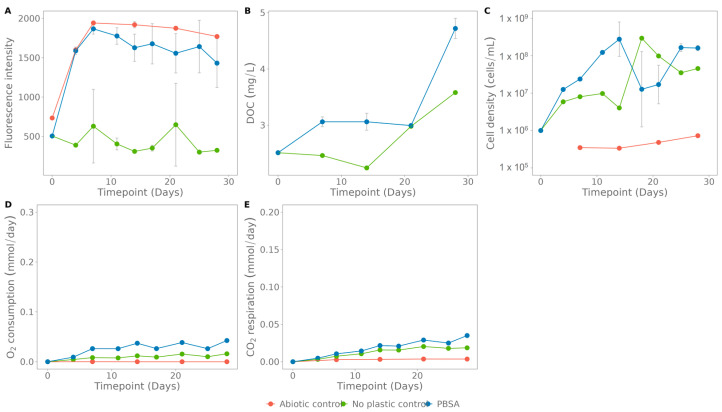
Assays for poly(butylene succinate-co-butylene adipate) (PBSA) biodegradation with the enriched seawater culture: (**A**) fluorescence, (**B**) dissolved organic carbon, (**C**) cell density, (**D**) O_2_ consumption rate, and (**E**) CO_2_ respiration rate. The abiotic control contains only plastic and NaN_3_, the no-plastic control contains only the enriched culture in medium. The standard deviation was calculated on three biological replicates.

**Figure A2:**
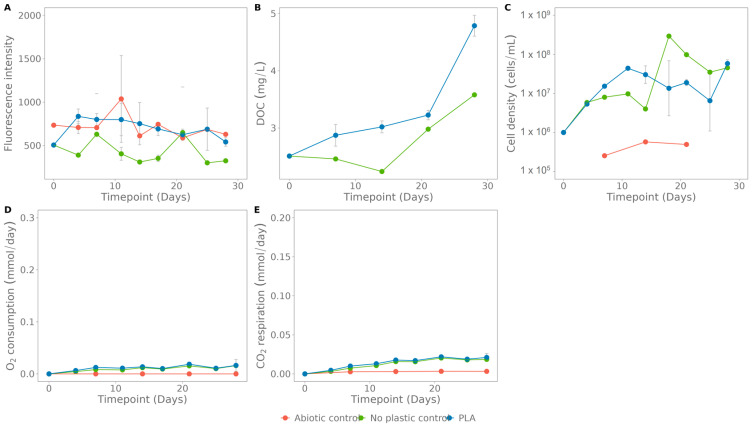
Assays for polylactic acid (PLA) biodegradation with the enriched seawater culture: (**A**) fluorescence, (**B**) dissolved organic carbon, (**C**) cell density, (**D**) O_2_ consumption rate, and (**E**) CO_2_ respiration rate. The abiotic control contains only plastic and NaN_3_, the no-plastic control contains only the enriched culture in medium. The standard deviation was calculated on three biological replicates.

Both PBSA (Figure A1) and PLA (Figure A2) did not show increased fluorescence compared to the control and only slight differences to the control in the other methods. This was expected for PLA, as it has been reported to be nonbiodegradable in the marine environment [4,46,47]. The results for PBSAindicate no biodegradation takes place during the experiment.

### 3.4. Case Study 2: Biodegradation of Plastic by Environmental Samples

#### 3.4.1. Coupure Water

**Figure 8:**
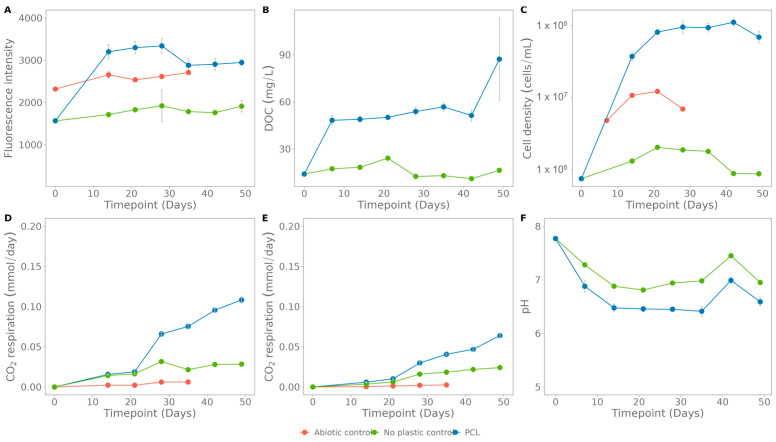
Assays for polycaprolactone (PCL) biodegradation in Coupure water: (**A**) fluorescence, (**B**) dissolved organic carbon, (**C**) cell density, (**D**) O_2_ consumption rate, (**E**) CO_2_ respiration rate, and (**F**) pH. The abiotic control contains only plastic and NaN_3_, the no-plastic control contains only Coupure water. The standard deviation was calculated on three biological replicates.

**Figure A3:**
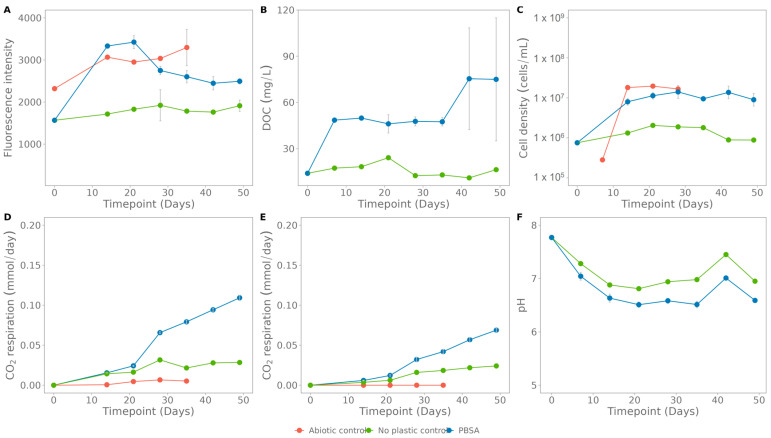
Assays for poly(butylene succinate-co-butylene adipate) (PBSA) biodegradation in Coupure water: (**A**) fluorescence, (**B**) dissolved organic carbon, (**C**) cell density, (**D**) O_2_ consumption rate, (**E**) CO_2_ respiration rate, and (**F**) pH. The abiotic control contains only plastic and NaN_3_, the no-plastic control contains only Coupure water. The standard deviation was calculated on three biological replicates.

**Figure A4:**
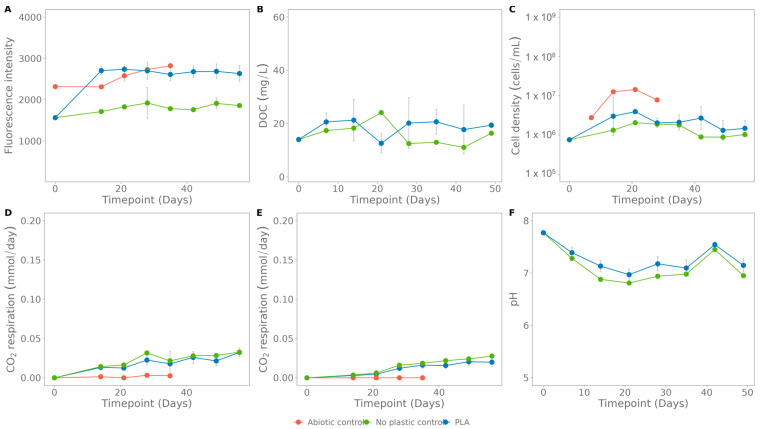
Assays for polylactic acid (PLA) biodegradation in Coupure water: (**A**) fluorescence, (**B**) dissolved organic carbon, (**C**) cell density, (**D**) O_2_ consumption rate, (**E**) CO_2_ respiration rate, and (**F**) pH. The abiotic control contains only plastic and NaN_3_, the no-plastic control contains only Coupure water. The standard deviation was calculated on three biological replicates.

Coupure water, with its indigenous microbiome, was incubated with the plastics PCL (Figure 8), PBSA (Figure A3) and PLA (Figure A4), containing carbon nanodots, to validate the potential of this biodegradation test for an environmental culture. PLA (Figure A4) did not show increased fluorescence compared to the control and only slight differences to the control in the other methods. For PCL, an increase in fluorescence at the start was observed, followed by a plateau phase (Figure 8A). This early increase was also observed in the DOC concentration (Figure 8B). The other measurements indicate activity of the community (Figure 8C–E). The pH drops in the beginning from 7.77 to 6.48 ± 0.07 and remains constant afterwards (Figure 8F). The trends in the results for PBSA are similar (Figure A3).

#### 3.4.2. Seawater

**Figure 9:**
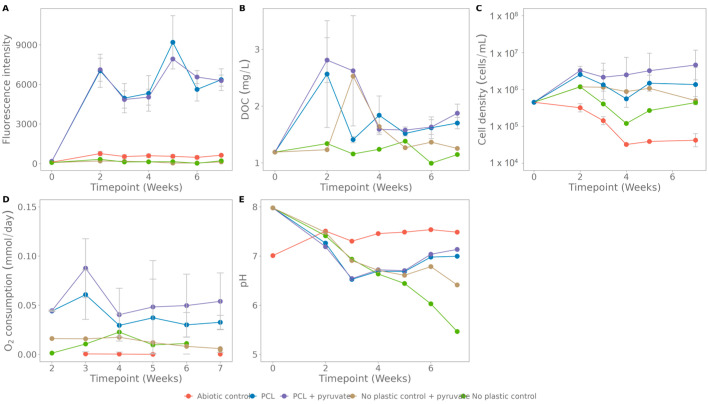
Assays for polycaprolactone (PCL) biodegradation in seawater: (**A**) fluorescence, (**B**) dissolved organic carbon, (**C**) cell density, (**D**) O_2_ consumption rate, (**E**) CO_2_ respiration rate. The abiotic control contains only plastic and NaN_3_, the no-plastic control contains only seawater. The standard deviation was calculated on three biological replicates.

In a similar setup as for Coupure water, PCL, with incorporated carbon nanodots, was incubated in seawater. The fluorescence pattern strongly increases in the first two weeks and stabilizes afterwards (Figure 9A). This early increase was also observed in the DOC concentration (Figure 9B). However, this organic carbon was consumed rapidly as levels dropped again in week 3 and 4. For the O_2_ consumption rate and pH (except for the no-plastic control, which is pure seawater) the values stabilize as well after four weeks (Figure 9D,E). Overall, no or very little growth was observed over seven weeks (Figure 9C). In this setup, some samples were supplied with an extra carbon source, pyruvate, to stimulate growth. It is observed that the addition of the extra carbon source did increase the O_2_ consumption rate and growth, but not the fluorescence nor the DOC.

## 4. Discussion

The potential of carbon nanodots for colorimetric plastic biodegradation tests was investigated in this study. The in-house carbon nanodots were confirmed to be photostable, biocompatible, soluble in water and have high fluorescence. These characteristics are ideal for the implementation of carbon nanodots in colorimetric tests for assessing biodegradation in both engineered systems and natural environments. It is noteworthy that this colorimetric assay only monitors the depolymerisation, the most difficult, rate-limiting step of plastic biodegradation [5,26].

PCL, with embedded carbon nanodots, was exposed to enzymatic degradation by CALB, upon which the fluorescent particles were released, analogous to the decreasing pH (Figure 6). This proof of concept indicated that the proposed method works [43,48,49]. As this colorimetric assay is a very reliable and fast technique for enzymatic degradation tests, the detection of microbial degradation was also investigated. In general, the method is clearly able to discriminate between microbial cultures that can depolymerise (PHBH, Figure 6), and cultures that cannot (PLA, Figure A2). As is confirmed by the data of PHBH, degraded by the enriched culture (Figure 6), the sensitivity of the method is as fast as CO_2_ respiration and O_2_ consumption. Moreover, the method is more selective, as CO_2_ respiration and O_2_ consumption can increase due to other (unwanted) carbon sources present, or due to mineralisation of leached residual mono- and oligomers [20,40,50]. This is, for example, demonstrated in Figure 8, where the increased CO_2_ respiration and O_2_ consumption rate do not correlate with an increase in fluorescence. In an attempt to discriminate which carbon sources are consumed, the dissolved organic carbon (DOC) was also monitored in these experiments, indicating the amount of soluble carbon during the incubation. By combining these different measurement methods, more insight into the biodegradation processes is possible. For example, for PCL degraded by the enriched culture (Figure 7). The CO_2_ respiration rate, O_2_ consumption rate and cell density are slightly increasing after day 10, while there is no increase in fluorescence, nor a decrease in DOC concentration during the test period. On the contrary, the DOC concentration increases towards the end of the incubation. Prior to this increase, the DOC concentration is, however, constant. Possibly, leached low molecular weight compounds are rapidly consumed by the bacteria, hence the increased growth and constant DOC value. Romera-Castillo et al. (2018) reported that 60% of DOC can be consumed in less than 5 days by marine bacteria [49,50]. After day 21, DOC levels increase and the CO_2_ respiration rate and cell density stagnate, possibly due to lack of nutrients that limit further carbon consumption. This assumption can be confirmed by a decrease in cell concentration from day 18 on. On the other hand, the control without plastic also has an increased DOC value, hence, this can also be due to carbon contamination while sampling.

PHBH, reported to be biodegradable in the marine environment [51,52], has an increase in the fluorescence over time when degraded by an enriched culture (Figure 6). Moreover, comparison of the different biodegradation methods indicates that growth of the culture, either on contaminated carbon or on leached low-molecular-weight compounds, precedes the biodegradation of the polymer. The lag phase in the fluorescence might be due to the types of enzymes. Possibly, the extracellular enzymes are inducible enzymes, of which the synthesis is only induced at the presence of a specific substrate. Hence, the concentration of the extracellular enzymes is too low for depolymerisation at the beginning of the incubation [16]. From the DOC concentration, it is observed that the concentration of oligomers increases. Assuming that 1 mg of released carbon nanodots corresponds to 1 g of degraded plastic (the carbon nanodots are homogeneously distributed in the polymer in a concentration of 1.072 mg/g plastic), it was estimated that 18.7 mg PHBH (8.5%) was depolymerised after 28 days of incubation, of which 29.2 mg/L (21%) was still present as DOC. The other 79% is mineralised by bacteria and converted to either cell biomass or CO_2_. Care should be taken, however, that the homogeneous distribution of the carbon nanodots is visually observed, not quantified. This method is thus certainly quantitative in amount of released carbon nanodots but rather qualitative in terms of plastic biodegradation and not accurate enough to determine exact biodegradation rates.

The results of the biodegradation tests in natural environments are, in general, hard to compare with the literature due to very variable plastics degradation rates reported [53]. For example, PCL, a fossil-based semicrystalline polyester, is reported to biodegrade by both aerobic and anaerobic microorganisms in natural environments [54]. However, when screening the literature, the degradability in seawater is debatable [9,54,55,56,57,58]. Many different degradation rates in marine systems are reported, ranging from no significant biodegradability and 0.5% biodegradation in 1 year to 100% degradation in 28 days, mainly due to different test conditions [53,54]. Additionally, the performed tests in this study only continued for 2 months, which might be too short in terms of biodegradation. Despite this, the slight increase in fluorescence for PCL in seawater from week 2 to week 7 (Figure 9, linear regression: fluorescence = 71,394*× time[weeks] + 6092.7, R^2^ = 0.0074) indicates slow biodegradation, which is supported by a slight increase in DOC. These results cannot be extrapolated easily [14], and no prediction on biodegradation on a longer time scale can be made.

In the degradation tests with PCL in Coupure water and seawater, a recurring fluorescence pattern is seen, starting with a steep increase in the first 14 days and followed by a plateau phase (Figure 8 and Figure 9). As the abiotic controls do not show a similar increase, this should be due to biological activity. This suggests a two-phase biodegradation: some easily accessible bounds in amorphous regions of the plastic that are very rapidly hydrolysed, after which the biodegradation rate decreases due to the crystalline structure and thus more difficult-to-access bounds [59].

The objective of this study was to obtain a reliable, less expensive, direct and fast biodegradation method. This colorimetric assay investigates the rate-limiting step of biodegradation, depolymerisation, but can be combined with other methods to retrieve information on mineralisation to CO_2_. However, to the best of our knowledge, most biodegradation techniques can only quantify part of the biodegradation process. Furthermore, depolymerisation is the most difficult step in the process and because of the high sensitivity of the carbon nanodots rapidly observed in this colorimetric test. As this method detects biodegradation directly, through depolymerisation of the polymer, no confounding results occur from biodegradation of other C sources, such as contaminated assimilable carbon or leached additives or low-molecular-weight compounds [20]. Compared to the indirect CO_2_ respiration and O_2_ consumption, this colorimetric method allows a well-aerated system, favourable for aerobic biodegradation. Compared to other techniques such as chromatography-based methods, this colorimetric test has an additional advantage. While chromatography requires the knowledge of the exact mono- and oligomers present and the development of new methods, this colorimetric technique is much easier and can be used without knowing the exact degradation pathway and intermediates of the plastic.

## 5. Conclusions

We can conclude from our results that different biodegradation test methods can give different indications on biodegradation activity. Therefore, the incorporation of controls is crucial for any biodegradation experiment, especially when using indirect measurement methods such as growth, O_2_ consumption and CO_2_ respiration.

The proposed colorimetric test, based on fluorescent carbon nanodots embedded in the polymer matrix, is an easy, sensitive, fast and most importantly direct technique to measure plastic biodegradation. The implementation of carbon nanodots as fluorescent markers adds additional advantages of biocompatibility and chemical and photostability to this technique compared to other fluorescent dyes. The plastic pieces containing in-house-made carbon-nanodots were successfully used as substrates for both enzymatic and microbial degradation tests. In conclusion, this colorimetric test is easy and fast to apply and furthermore a good fit to screen, in high throughput, the depolymerisation of plastics in natural environments or under different conditions in the lab.

## Data Availability

The data presented in this study are available in the Figures and Appendix A.
